# Pathogenesis of Celiac Disease and Other Gluten Related Disorders in Wheat and Strategies for Mitigating Them

**DOI:** 10.3389/fnut.2020.00006

**Published:** 2020-02-07

**Authors:** Natasha Sharma, Simran Bhatia, Venkatesh Chunduri, Satveer Kaur, Saloni Sharma, Payal Kapoor, Anita Kumari, Monika Garg

**Affiliations:** Agri-Food Biotechnology Laboratory, National Agri-Food Biotechnology Institute, Mohali, India

**Keywords:** wheat, gluten, celiac disease, non-celiac gluten sensitivity, wheat allergy, gluten-free diet

## Abstract

Wheat is a major cereal crop providing energy and nutrients to the billions of people around the world. Gluten is a structural protein in wheat, that is necessary for its dough making properties, but it is responsible for imparting certain intolerances among some individuals, which are part of this review. Most important among these intolerances is celiac disease, that is gluten triggered T-cell mediated autoimmune enteropathy and results in villous atrophy, inflammation and damage to intestinal lining in genetically liable individuals containing human leukocyte antigen DQ2/DQ8 molecules on antigen presenting cells. Celiac disease occurs due to presence of celiac disease eliciting epitopes in gluten, particularly highly immunogenic alpha-gliadins. Another gluten related disorder is non-celiac gluten-sensitivity in which innate immune-response occurs in patients along with gastrointestinal and non-gastrointestinal symptoms, that disappear upon removal of gluten from the diet. In wheat allergy, either IgE or non-IgE mediated immune response occurs in individuals after inhalation or ingestion of wheat. Following a life-long gluten-free diet by celiac disease and non-celiac gluten-sensitivity patients is very challenging as none of wheat cultivar or related species stands safe for consumption. Hence, different molecular biology, genetic engineering, breeding, microbial, enzymatic, and chemical strategies have been worked upon to reduce the celiac disease epitopes and the gluten content in wheat. Currently, only 8.4% of total population is affected by wheat-related issues, while rest of population remains safe and should not remove wheat from the diet, based on false media coverage.

## Introduction

Cereal crops are recognized as a major food source for mankind. Wheat, rice and barley are some of the major cereal crops grown worldwide. Cereals are staple food for human nutrition and are considered as a major source of calories for human (1). The dietary components in cereals such as lipids, carbohydrates and proteins, play an instrumental role in processing and nutritional quality for food and feed. Among cereals, wheat is one of the major staple crops across the world and is unique due to its special bread-making properties. The estimated global wheat production for 2016 was 749.46 million metric tons (2). Furthermore, the global demand of wheat has increased incredibly after industrialization and urbanization due to its bread making properties and ability of being processed into different food products (3). The most commonly used wheat types are durum or tetraploid wheat (A and B genome) and hexaploid wheat (A, B, and D genome). Many species of wheat together make up the genus *Triticum*, amongst which the most widely used and grown is the hexaploid wheat (*Triticum aestivum* L. AABBDD) (4).

Wheat seed storage proteins are very important in determining the end products as they impart viscoelasticity and extensibility to dough which enables formation of a wide range of products such as bread, pasta, noodles, cakes, and pastries (3, 5). Seed storage proteins constitute about 8–15 percent of total flour weight and can be classified into albumins, globulins, gliadins, and glutenins on the basis of their solubility. Of these fractions, gliadins and glutenins constitute the gluten proteins and are stored together with starch in endosperm of the seed. Both gliadins and glutenins are involved in building the gluten polymer and determining bread-making properties of wheat (6). But, gluten present in wheat is the major factor responsible for causing certain disorders and allergies in some individuals. A wide variety of people are incapable to tolerate wheat consumption due to harmful immune response to gluten proteins present in wheat. Hence, despite of such large consumption of wheat worldwide, there are cases reported which show intolerance toward it (7). The most common wheat-related disorders associated with gluten ingestion are celiac disease (CD) and non-celiac gluten-sensitivity (NCGS), which result in impaired quality of life and significant morbidity in individuals (8). Wheat allergy is another condition arising from contact, inhalation or ingestion of wheat and is associated with gluten, other wheat proteins and carbohydrates present in wheat particularly fermentable, oligo, di, monosaccharides, and polyols (FODMAPs). Specific clinical manifestations can be observed in each of these disorders with some peculiar immunogenic pathways involved in their development (9). Adherence to gluten free foods is the only available remedy for patients with CD and NCGS. This manuscript provides detailed insight into the pathogenesis and mechanisms of gluten related disorders, particularly CD along with NCGS and wheat allergy; and different strategies to lower down wheat toxicity and gluten content in wheat.

## Components of Wheat Involved in Intolerance

Different components of wheat which are responsible for eliciting immune response and gastrointestinal symptoms in certain individuals are:

### Gluten

Gluten is the main storage protein found in wheat, rye and barley; and is important for dough formation (10). Gluten is classified as: (a) high molecular weight glutenin subunits (HMWGS); (b) low molecular weight glutenin subunits (LMWGS); (c) the S-poor prolamins (omega [ω]-gliadins); and (d) S-rich prolamins which include alpha (α), beta (β), and gamma (γ) gliadins (11–13). Gluten composition varies between both species as well as cultivars.

Glutens contain high contents of proline-rich polypeptide residues which make them resistant to proteolytic degradation by gastric, pancreatic, and intestinal juices containing digestive proteases (8, 14–17). When these proteins are consumed by genetically susceptible individuals, a cascade of immune reactions is triggered, which result in damage to the intestinal lining resulting in CD. Gluten is also responsible for causing other wheat related disorders such as NCGS, wheat allergy and contact urticaria (8, 9). The most widely prevalent of all is CD.

### α-Amylase/Trypsin Inhibitors (ATIs) and Lectins

ATIs and lectins comprise of 2–4% of total proteins in modern hexaploid wheat. Wheat ATIs are disulphide linked, compact albumin proteins found in the endosperm of plant seeds and are resistant to degradation by the proteases (18). These proteins regulate starch metabolism during seed development and germination, and aid in providing defense to plants against parasites and insects (19, 20). ATIs have recently been implicated in wheat sensitivity. ATIs trigger innate immune response by activating toll-like receptor (TLR) 4 on myeloid cells and antigen presenting cells such as monocytes, macrophages, and dendritic cells in intestinal mucosa to produce inflammatory response by producing cytokines and chemokines, *viz*. interleukin (IL)-8, tumor necrosis factor (TNF)-α, or monocyte chemotactic protein-1 (21). Once antigen presenting cells are activated by ATIs, they migrate to peripheral lymph nodes and further enhance the ongoing immune response (22). ATIs mainly produce non-intestinal symptoms in NCGS (23–25) and also act as primary allergens in Baker's asthma (18). Experimental evidences show that ATIs serve as adjuvants in intestinal inflammatory diseases in mice (26).

Lectins are carbohydrate-binding proteins present in plants that provide defense to plants against pathogens (27, 28). Wheat germ agglutinin is a specific type of lectin which is described extensively in the literature for inducing adverse health effects. It binds to gut epithelium, damages intestinal cells, and results in reduced absorption of nutrients in the gut (29, 30).

### FODMAPs

FODMAPs are short-chain carbohydrates comprising of fructans and galacto-oligosaccharides, and are present naturally in many foods in various forms *viz*. lactose in milk, free fructose in fruits like pears and apples, fructans in wheat and onions, galacto-oligosaccharides in legumes and sugar polyols *viz*. sorbitol and mannitol in stone fruit, some vegetables, and fermented foods (31). In wheat, FODMAPs *viz*. fructans, galacto-oligosaccharides, and mannitol are present. Human body, lacks enzymes for the breakdown of FODMAPs and hence these get absorbed slowly in the small intestine and pass undigested to reach large intestine where these are rapidly fermented by gut bacteria which produce gas and cause intestinal walls to stretch (32, 33). In most people, this process is asymptomatic, but in patients with irritable bowel syndrome and inflammatory bowel disease, this process is problematic and can produce various symptoms including flatulence, bloating, stomach pain, constipation, or diarrhea (34–36). Biesiekierski et al. (29) observed that removal of dietary FODMAPs caused improvement in gastrointestinal symptoms in 37 people with NCGS and irritable bowel syndrome.

## Wheat Related Disorders

**Figure 1:**
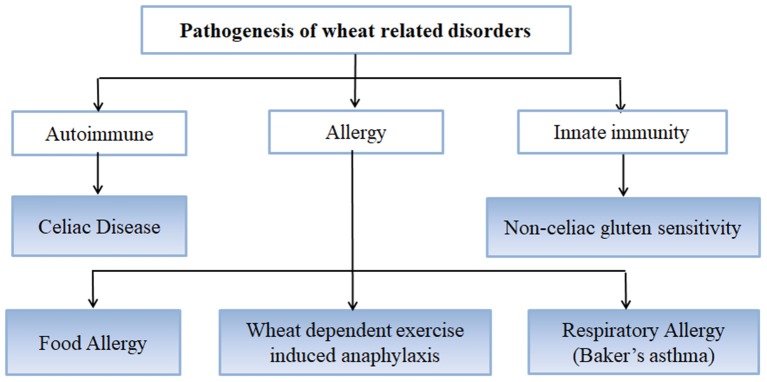
Immune reactions involved in wheat related disorders.

Wheat is the most important cereal crop worldwide and has been used as an important staple crop for centuries but in certain individuals and genetically predisposed people, it causes certain disorders and conditions arising mainly from the gluten fraction [Figure 1; (37)]. CD is caused by gluten proteins specifically gliadins but the problem lies not just with gluten protein but with other non-prolamins components of wheat as well which results in the onset of some other conditions as well (38). These include NCGS in which patients experience symptoms similar to CD but these get resolved when gluten is removed from the diet. However, patients do not test positive for CD (39). Other non-celiac wheat response is wheat allergy, which includes Baker's asthma, a respiratory allergic response caused by exposure to wheat flour dust (40–42); food allergy which is IgE-mediated immune response caused by wheat ingestion; and wheat-dependent exercise-induced anaphylaxis (WDEIA) which is exercise induced wheat allergic response (30). The most common wheat related disorders are:

### CD

**Figure 2:**
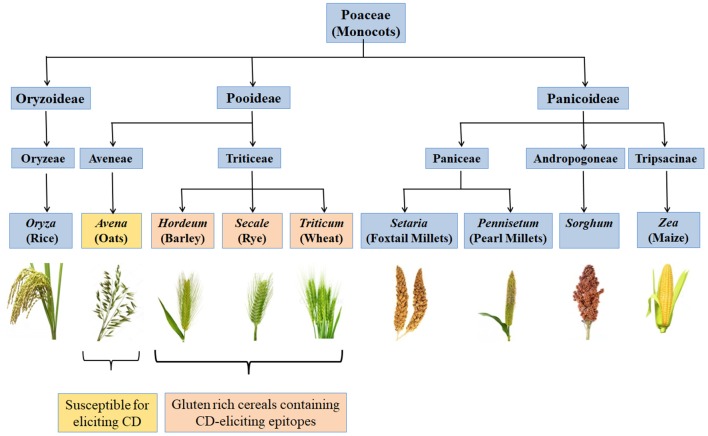
Classification of monocots and their CD eliciting potential. Gluten rich cereals *viz*. wheat, rye, and barley belonging to family *Triticeae* and containing CD-eliciting epitopes (pink boxes). Some cereals like oat are susceptible for eliciting CD (yellow box) whereas, many other cereals are safe for CD patients (blue boxes).

CD is a chronic immune-mediated enteropathy of the small intestine that develops in genetically susceptible individuals by exposure to gluten proteins found in certain cereals *viz*. wheat, rye and barley (43, 44). All these are close relatives of wheat, while distantly related species do not elicit CD. There are controversial studies related to involvement of oats in causing CD (45–48). Though oats do not contain gluten, these contain a small percentage (10–15% total protein content) of similar proline rich storage proteins called “avenins.” Few CD epitopes with different structures have been identified in oats which may cause intolerance in some individuals at low intensity (49, 50). But more serious problem is the production of pure oats free from gluten containing cereals in a conventional production chain as there are high chances of contamination with wheat, rye and barley during seed sowing, cultivation, harvesting, milling, and processing of oats (51). Figure 2 shows various gluten-rich cereals capable of eliciting CD immunogenicity in patients. Both glutenins and gliadins have certain amino acid sequences that act as epitopes for CD (52) and are termed as immunogenic peptides, antigenic peptides, T-cell epitopes, CD eliciting epitopes, or toxic peptides (53). These CD eliciting peptides resist degradation in gastrointestinal tract due to high content of amino acids *viz*. proline and glutamine.

CD represents a chronic inflammatory condition affecting small intestine and jejunum, resulting in villous atrophy in intestine. The villi in small intestine get flattened and the surface area for nutrient absorption is highly reduced which leads to malnutrition, vitamin and mineral deficiencies and other gastrointestinal symptoms such as abdominal discomfort, bloating, loose bowel movements, and nausea (54, 55). Untreated patients develop risk of some chronic conditions and non-gastrointestinal symptoms such as anemia, osteoporosis, fatigue, infertility, eczema, and refractory CD which is associated with developing lymphoma (44, 56). Symptoms of CD are highly variable and may occur at any age. Diseases like type I diabetes, Hashimoto's thyroiditis, Grave's disease, Sjogren's syndrome, Down syndrome, Turner syndrome, primary biliary cirrhosis, and neurologic disorders like unexplained peripheral neuropathy, epilepsy, and ataxia are also sometimes associated with CD (57–63).

The global prevalence of CD was found to be 1.4% in 275,818 individuals studied using serological antibodies. According to biopsy based analysis, its prevalence was found to be 0.7% in ~1,40,000 individuals studied (53). In different countries, the range of disease varied as 0.5% in Africa and North America, 0.4% in South America, 0.6% in Asia, and 0.8% in Europe. The prevalence of disease varies with age, sex and presence of other autoimmune disorders (53). Currently, adhering to gluten free foods is the only option for individuals with CD (37, 64, 65).

#### CD Epitopes

**Figure 3:**
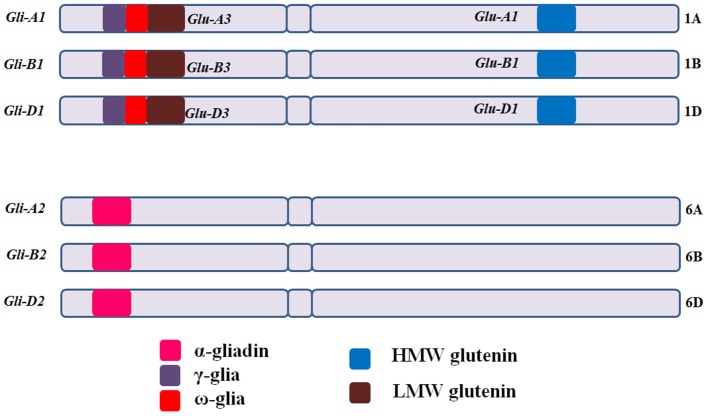
Gliadin and glutenin loci in *Triticum aestivum* (AABBDD), 2n = 6x = 42. The *Gli-A1, Gli-B1*, and *Gli-D1* are located on the short arm of homoeologous group −1 chromosomes and comprise mainly of ω and γ-gliadins, while *Gli-A2, Gli-B2*, and *Gli-D2* are located on short arm of group −6 chromosomes and comprise mainly of α-gliadins. HMWGS *viz*. *Glu-A1, Glu-B1*, and *Glu-D1* are located on long arm of group −1 chromosomes, whereas, LMWGS *viz*. *Glu-A3, Glu-B3*, and *Glu-D3* are located on the short arm of group −1 chromosomes. Gli, gliadin; Glu, glutenin.

CD eliciting epitopic sequences of glutens from hexaploid bread wheat (*T. aestivum* L.) are present in mainly six loci, with *Gli-A1, Gli-B1*, and *Gli-D1* located on short arms of group 1 chromosomes (1AS, 1BS, and 1DS) and *Gli-A2, Gli-B2*, and *Gli-D2* on the short arms of group 6 chromosomes (6AS, 6BS, and 6DS) [Figure 3; (11, 66)]. *Gli-1* loci contain genes coding for γ, ω, or δ gliadins, whereas, *Gli-2* loci contain genes coding for α-gliadins (66–68). HMWGS are encoded by three homoeologous loci (*Glu-A1, Glu-B1*, and *Glu-D1*) on the long arms of group 1 chromosomes, while LMWGS are encoded by *Glu-A3, Glu-B3*, and *Glu-D3* loci on the short arms of group 1 chromosomes [Figure 3; (68, 69)].

Till date many T-cell epitopes have been studied for inducing immunogenicity of CD. Both glutenins and gliadins are responsible for this disease. The major epitopes from wheat responsible for eliciting CD immunogenicity are listed in Table 1. All three α/β, ω and γ-gliadins are immunogenic for CD (52) but the 33-mer peptide sequence (LQLQPFPQPQLPYPQPQLPYPQPQLPYPQPQPF) of α-gliadins is found to be the most potent stimulator of T-cells and is the most immunogenic amongst all (52, 72, 73). The high importance of the 33-mer sequence in CD is evident by the production of two monoclonal antibodies (A1 and G12) against it (74). This 33-mer peptide fragment of gliadin α2 (57–67, 69–90) contains 6 partially overlapping copies of three highly immunogenic T cell epitopes, *viz*. PFPQPQLPY (DQ2.5-glia-α1a, 1 copy), PYPQPQLPY (DQ2.5-glia-α1b, 2 copies), and PQPQLPYPQ (DQ2.5-glia-α2, 3 copies) (70, 75, 76). The 33-mer sequence is rich in proline and glutamine residues and is a stimulator of T-cells after deamidation by the enzyme tissue transglutaminase (TG2) (77).

**Table 1:** Major epitopes from wheat prolamines involved in CD immunogenicity.

**S.No**.	**Epitope**	**Protein source**	**HLA-complex**	**Peptide sequence**
1.	Gli-α1a	α-gliadins	HLA-DQ2.5	PFPQP**Q**LPY
2.	Gli-α1b	α-gliadins	HLA-DQ2.5	PYPQP**Q**LPY
3.	Gli-α2	α-gliadins	HLA-DQ2.5	PQP**Q**LPYPQ
4.	Gli-α3	α-gliadins	HLA-DQ2.5	FRP**Q**QPYPQ
5.	Gli-γ1	γ-gliadins	HLA-DQ2.5	PQQSFP**Q**QQ
6.	Gli-γ2	γ-gliadins	HLA-DQ2.5	QP**Q**QPAQL
7.	Glia-γ3	γ-gliadins	HLA-DQ2.5	QQP**Q**QPYPQ
8.	Glia-γ4a	γ-gliadins	HLA-DQ2.5	SQP**Q**Q**Q**FPQ
9.	Glia-γ4b	γ-gliadins	HLA-DQ2.5	PQP**Q**Q**Q**FPQ
10.	Glia-γ4c	γ-gliadins	HLA-DQ2.5	QQP**Q**QPFPQ
11.	Glia-γ4d	γ-gliadins	HLA-DQ2.5	PQP**Q**QPFCQ
12.	Glia-γ5	γ-gliadins	HLA-DQ2.5	QQPFP**Q**QPQ
13.	Glia-ω1	ω-gliadins	HLA-DQ2.5	PFPQP**Q**QPF
14.	Glia-ω2	ω-gliadins	HLA-DQ2.5	PQP**Q**QPFPW
15.	Glut-L1	LMW-glutenins	HLA-DQ2.5	PFS**Q**Q**Q**QPV
16.	Glut-L2	LMW-glutenins	HLA-DQ2.5	FSQQQ**Q**SPF
17.	Glut-L1	LMW-glutenins	HLA-DQ2.2	PFS**Q**Q**Q**QPV
18.	Gli-α1	α-gliadins	HLA-DQ8	**Q**GSFQPSQ**Q**
19.	Glia-γ1a	γ-gliadins	HLA-DQ8	**Q**QPQQPFPQ
20.	Glia-γ1b	γ-gliadins	HLA-DQ8	**Q**QPQQPYP**Q**
21.	Glut-H1	HMW-glutenins	HLA-DQ8	QGYYPTSPQ
22.	Gli-α1	α-gliadins	HLA-DQ8.5	**Q**GSFQPSQ**Q**
23.	Gli-γ1	γ-gliadins	HLA-DQ8.5	PQQSFP**Q**Q**Q**
24.	Glut-H1	HMW-glutenins	HLA-DQ8.5	QGYYPTSPQ

*Table adapted from Sollid et al. (70) and Shewry and Tatham (71). Table shows different CD-eliciting epitopes from α, γ, ω gliadins; LMWGS and HMWGS capable of eliciting T-cell mediated immune response in CD patients. Amino acids shown in bold in peptide sequences depict glutamine residues targeted for deamination by the enzyme T2G. Gli-α, α gliadin; Gliγ, γ gliadin; Gli-ω, ω gliadin; Glut-L, LMWGS; Glu-H, HMWGS*.

Estimated copy number of α-gliadins ranges from 25 to 150 copies per haploid genome and consists of a highly diverse and complex gene family (78). α-gliadins from D-genome (specified by *Gli-D2*) are the most immunogenic while those from B-genome (specified by *Gli-B2*) are least immunogenic as these contain very few CD epitopes (44, 71, 79–82). After α-gliadins, γ-gliadins are found to be most immunogenic and their copy number ranges from 15 to 40 (83). HMWGS and LMWGS also contain CD epitopes but in less amount and present low immunogenicity (44, 71). Also, single or multiple amino acid substitutions in natural epitopic sequences result in the lack of immunogenicity. For example, proline to serine substitution in epitope core position p3 or p8, or proline to alanine substitution in epitope core position p5 in PFPQPQLPY sequence of DQ2.5 Gli-α1a resulted in lack of T-cell stimulation and reduced immunogenicity. Similarly, serine to phenylalanine substitution at p3, or glutamine to arginine substitution at p5 in QGSFQPSQQ sequence of DQ8.5 Gli-α1 completely removed the immunogenic potential of the epitope (84).

#### Mechanism of CD Toxicity

CD is an auto-immune disorder and its pathogenicity results from the interaction of gluten with genetic and environmental factors which initiate an immune response in the body. At genetic level, CD occurs in genetically predisposed individuals carrying specific major histocompatibility complex haplotype DQ2 or DQ8 in Human Leukocyte Antigen (HLA) in certain individuals (54, 85–87). In a study by Ciccocioppo et al. (88), it was found that gluten peptides involved in CD were able to specifically stimulate HLA-DQ2 or HLA-DQ8-restricted T-cell clones isolated from jejunal mucosa or peripheral blood of celiac patients. The HLA-DQ2 and HLA-DQ8 are cell surface receptors located on antigen presenting cells and contain positively charged pockets with a preference for binding negatively charged particles; and thus, have a strong binding affinity to these negatively charged gluten molecules (89). Over 95% of the affected CD patients express HLA-DQ2 molecules and the remaining express HLA-DQ8 (90, 91). Further in a process of CD, zonulin, a human protein and a modulator of intestinal tight junctions is involved in a reversible regulation of intestinal permeability (92). Its upregulation due to gluten exposure disrupts the integrity of tight junctions between epithelial cells of small intestine and increases the paracellular movement of protease resistant gluten fragments (93, 94). As a result, the undigested gluten peptide fragments pass through epithelial barrier of small intestine and enter lamina propria where these are deaminated by the enzyme tissue transglutaminase (TG2) which converts glutamine to glutamate thereby imparting negative charge to gluten fragments (95). Further, the activity of the enzyme TG2 is dependent on spacing between glutamine and proline. Glutamine in the sequence QXP is modified by the enzyme but in the sequence QXXP and QP it remains unmodified (X = any amino acid) (96). Deamination of gluten peptides by TG2 is the key pathogenic event that increases gliadin immunogenicity in CD and enhances the severity of disease (97). TG2 plays an important role in CD pathogenesis and anti-TG2 antibodies are used as the markers for CD diagnosis (95). The enzyme TG2 is located on the brush border epithelia of the small intestine or in extracellular space of sub-epithelial region (98). TG2 exists in inactivated form under normal oxidative conditions; but gets activated extracellularly under reducing conditions created by inflammatory response during CD (99). The negatively charged peptides produced through deamination by TG2 bind to the positively charged amino acids on HLA-DQ2 and DQ8 molecules and trigger adaptive as well as innate immune response in CD patients (88, 100). Adaptive immune response begins with the presentation of undigested gluten peptides by HLA molecules on antigen presenting cells to CD4+ T-cells in lamina propria. The recognition and binding of T-cell receptors to specific HLA-gliadin complexes lead to the production of high levels of pro-inflammatory cytokines dominated mainly by interferon (IFN)-γ (65, 101, 102). These cytokines either induce T-helper 1 cells to produce IL-15 and IL-21 which result in the activation of cytotoxic CD8+ intra epithelial lymphocytes (IELs) and promote CD8+ T-cell cytotoxic activity and contributes to intestinal lesions, inflammation and intestinal mucosal damage (95, 103) or T-helper 2 cells to cause B-lymphocytes differentiation for the secretion of anti-gliadin, anti-TG2, and anti-endomysium (EM) antibodies which are considered as the key characteristics of active CD (65, 104). An increased density of CD8+ IELs is also considered as an important characteristic of CD (90, 105). These immune responses disappear when gluten is excluded from the diet (65). Some gliadin peptides bind to TLR2 receptors which result in increased IL-1 production, through the mediation of MyD88, a key protein responsible for mediating the release of zonulin in response to gluten ingestion (106). Recently it has been claimed that the presence of HLA is not the only factor responsible for the onset of CD (107). The genome-wide association studies have identified 39 non-HLA loci affecting CD (108).

Several environmental factors also influence the occurrence of CD. Feeding patterns in the first year of life and the time at which gluten intake is initiated during infancy also determines the susceptibility to disease (64, 105). The initial intake of gluten before 4 months of age contributes to disease susceptibility whereas administration of gluten after 7 months denotes marginal risk to disease. Gluten intake along with breast feeding in infants reduces the risk of the disease (109). Again, this factor is just one aspect that can contribute to celiac susceptibility. Some recent studies have claimed that the potential viral infections caused by rotavirus might play an important role in the activation of this disease (110). The individuals having CD are found to have rotavirus infection but there is no confirmed proof for this aspect.

#### Diagnosis of CD

**Figure 4:**
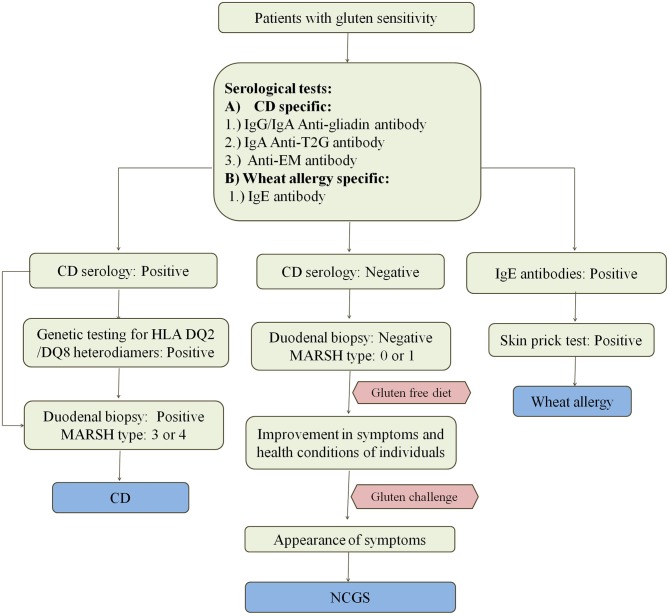
Diagnosis of wheat related disorders in patients. Figure shows different parameters for diagnosis of CD, NCGS, and wheat allergy. CD patients are confirmed on the basis of serological tests which include the detection of anti-gladin, anti-T2G, and anti-EM antibodies in the blood serum of patients. CD can be further confirmed by positive genetic testing for HLA DQ2/DQ8 heterodimers and MARSH type 3–4 on the basis of duodenal biopsy. Wheat allergy is detected on the basis of presence of IgE antibodies in the blood serum of patients and confirmed on the basis of positive skin prick test. NCGS is detected on the basis of negative serological tests for CD in patients showing gluten sensitivity, gastrointestinal symptoms and MARSH type 0–1. NCGS in patients is further confirmed by observing improvement in symptoms upon intake of gluten-free diet. If symptoms reappear upon gluten challenge, then NCGS is confirmed.

CD enteropathy occurs in 1.4% of population but remains undiagnosed in most of the cases despite having evidence of increasing rates of diagnosis (111). Various methods for the diagnosis of CD and other wheat related disorders are given in Figure 4. Study by Jansen et al. (112) showed that in a population of around 4,500 children aged around 6 years, 61% had sub-clinical CD when screened with TG2 IgA antibody. At this time, there are no confined tests that can precisely confirm the cause and occurrence of disease. Since there are so many factors controlling the occurrence of disease, there is a need for the standard test for its confirmation. Most physicians these days rely upon serological tests using specific antibodies or prefer the use of biopsies for checking the intestinal damage caused by the disease. Flow cytometric analysis of IELs is very helpful for the diagnosis of CD in few cases where serological tests and duodenal biopsies do not work (113).

Serological testing is performed using anti-gliadin or anti-deaminated gliadin antibodies, anti-TG2 antibodies and anti-EM antibodies (114–119). Serological testing is performed on patients with some specific symptoms which include chronic diarrhea resulting in malabsorption of nutrients, iron deficiency anemia and deficiency of folates, vitamin E, D, K which result in apparent weight loss, osteoporosis, hypocalcemia, and unexplained elevation of transaminases (65, 118, 120–122).

In a study done using deaminated antibodies, it was found that anti-deaminated gliadin antibodies are better than conventional gliadin antibodies (123). The next in the list are TG2 specific antibodies which are found in serum as IgA and IgG isotypes, and are autoantigens for CD (124). Assaying for TG2-specific IgA is the most common clinical practice used in case of CD because of its highest specificity and sensitivity among all other methods (65, 125, 126). The sensitivity of these antibodies was tested on both humans as well as guinea pigs. Human TG2 antibodies were diagnosed in 64 out of 65 patients as compared to 58 out of 65 patients in case of guinea pigs. Hence, human TG2 antibodies are a better way to diagnose CD as compared to any other method (125). The next are anti-EM antibodies which were first studied on monkey esophagus. These antibodies react with EM of smooth muscles (127, 128). The specificity and sensitivity of IgA antibodies are 99 and 95%, respectively. A positive result of IgA EM antibodies is an indication of non-atrophic intestinal lesions (116). Prior to these serological methods, detection of only histological changes in small intestinal such as villous atrophy and presence of inflammatory markers were considered essential for the diagnosis of CD.

Genetic testing for CD follows the procedure for HLA-typing, but this test has low specificity. Determination of HLA-DQ2 and HLA-DQ8 types is useful along with histological findings (129). HLA-DQ2 and HLA-DQ8 molecules are associated with ~95 and 5% CD patients, respectively (59).

Serological testing by the use of anti-gliadin antibodies, TG2 or EM specific antibodies along with HLA-typing sometimes may not be successful and it cannot be considered as the confirmatory test. Individuals who are positive for serological testing are further confirmed with the help of biopsy. Sero-negative individuals also have to undergo biopsy if they have signs and symptoms highly suspicious for CD as none of the available serological tests have the sensitivity of 100% (130). Duodenal biopsy is the most preferred choice for the diagnosis of CD (131). The histological analysis of disease is performed using Mucosal Algorithmic Rules for Scoring Histology (MARSH) classification as discussed further. Endoscopic observation includes scalloping, fissuring, and the reduced villi folds in intestine. The pathologic lesion is a characteristic feature of disease. It is characterized by flat intestinal mucosa with infiltration of lymphocytes. Other symptoms include crypt hyperplasia and villous atrophy. Patient may suffer from anemia due to malabsorption (132). Sometimes due to poor results of both serological tests and biopsy, esophago gastro-duodenoscopy is done in CD subjects (116).

##### MARSH classification for histological diagnosis of CD

The recognition of histopathology of CD is done using MARSH classification system which describes the stages of damage in the small intestine and abnormalities in celiac mucosa during the progression of CD as seen under the microscope. MARSH classification system is useful because it avoids the misinterpretation of histo-pathological results of duodenum and hence, increases the sensitivity of the system (133). The modern MARSH classification system is given in Table 2.

**Table 2:** Modern MARSH classification system.

**MARSH grade**	**Significance**	**IEL count/100 enterocytes (duodenum)**	**IEL count/100 enterocytes (jejunum)**
MARSH type 0	Normal villi, Normal crypt hyperplasia	<30	<40
MARSH type I	Lymphocytic infiltration of villous epithelial layer	>30	>40
MARSH type II	Lymphocytosis along with crypt hyperplasia and high mitotic activity. Villous height/ crypt ratio decreases below normal value of 3–5	>30	>40
MARSH type IIIa	Partial villous atrophy with villi height/crypt ratio <1	>30	>40
MARSH type IIIb	Subtotal villous atrophy	>30	>40
MARSH type IIIc	Total villous atrophy with no distinguishable digitations	>30	>40
MARSH type IV	Describes a rare histologic finding of a flat atrophic mucosa which signifies irreversible injury caused by chronic inflammation	>30	>40

*Table adapted from Siriweera et al. (134) and Pena (135). Table shows the MARSH classification system for the detection of CD in patients on the basis of duodenal biopsy. MARSH type 0 represents villi of the normal healthy individual with IEL count of <30 per 100 enterocytes in duodenum as well as jejunum; whereas, increased MARSH type represents increased intestinal and villi damage. MARSH type IV represents completely damaged and eroded flat villi in patients. CD patients have MARSH type III or IV. IEL, intra epithelial lymphocytes; MARSH; Mucosal Algorithmic Rules for Scoring Histology*.

In this classification system, a five-point scoring system is given by Catassi and Fasano (136), which is called 4 out of 5 rule system. Individuals who satisfy any four of these are considered to be susceptible for CD. But this criterion is not widely used by clinicians since the gain in sensitivity in this case is at the cost of its specificity. According to this, the five points are (i) symptoms of CD such as diarrhea, weight loss and iron deficiency anemia, (ii) positive CD serologies at high titer, (iii) presence of a DQ2 or DQ8 haplotype, (iv) characteristic histopathologic findings, and (v) serological or histological response to the gluten-free diet.

#### Strategies for Lowering Down CD Epitopes

Following a life-long gluten-free diet is very difficult for CD patients. As a result, different strategies and therapies have been being discovered and explored to reduce the potential toxicity of gluten in CD (137). CD epitopes present in wheat can be lowered through various molecular biology, biotechnology, plant breeding, microbial, enzymatic, nano-technology, chemical, and pharmaceutical approaches (137–142).

Gene silencing by RNA-interference (RNAi) technology has been adopted for the successful down-regulation of α, γ, ω gliadins, and HMWGS in different studies (17, 143–146). Other non-transgenic approaches for lowering gluten epitopes in wheat include engineering gliadin epitopes in wheat with CRISPR/Cas9 (147); creating wheat deletion lines lacking α-gliadins on short arm of 6D chromosome (148–150); and wheat deletion lines lacking ω, γ gliadins, and LMWGS on short arm of chromosome 1D (151). Breeding hexaploid wheat varieties with less immunogenic wild relative of wheat *Agropyron elongatum* resulted in a significant reduction in immunogenic α-gliadin epitopes in subsequent generations (152, 153). In another study by Vita et al. (154), the ω-secalin gene encoding decapeptide QQPQRPQQPF from wheat-rye 1BL.1RS translocation line prevented K562 cells agglutination and mucosal cell immune activation in the presence of toxic gliadin epitopes. Another approach used in the reduction of CD-eliciting immunogenic gluten epitopes in wheat involves the use of microorganisms for the gluten hydrolysis, which occurs due to the presence of enzyme prolyl endopeptidases. Different microorganisms such as *Aspergillus niger, Flavobacterium meningosepticum, Sphingomonas capsulate*, and *Myxococcus Xanthus* have been used for gluten hydrolysis (155, 156).

The most widely reported strategy in lowering CD pathogenicity involves the use of probiotics which grow optimally under the pH range present in gastrointestinal tract and exert protective effects on gut mucosa and microbiota (157–159). Some probiotics have the potential to digest gluten polypeptides or help in their alteration or breakdown into simpler non-immunogenic peptides. The commercialized probiotic preparation VSL#3 comprising of consortium of eight bacterial strains (*Bifidobacterium breve, B. longum, B. infantis, Lactobacillus plantarum, L. acidophilus, L. casei, L. delbruecki* subsp*. bulgaricus*, and *Streptococcus thermophilus*) (160) was found to hydrolyze gliadin epitopes in wheat flour including immunogenic 33-mer peptide sequence during prolonged fermentation (161, 162). Smecuol et al. (163) observed that probiotic administration of *B. infantis* Natren Life Start superstrain in CD patients resulted in the marked improvement in digestion and reduction in constipation after 3 weeks from the beginning of treatment. Other examples of the use of probiotic strains for protecting epithelial cells of small intestine against cellular damage and reducing the levels of inflammatory cytokines have been given in Table 3.

**Table 3:** Strategies for lowering celiac disease epitopes.

**S.No**.	**Approach**	**Target**	**Features**	**Remarks**	**References**
**(A) Genetic Modification**					
1.	RNAi	Prolamins: α, γ, ω gliadins	90% reduction in prolamins	Gene silencing	(17)
2.	RNAi	HLA DQ2-α-II, DQ2-γ-VII, DQ8-α-I and DQ8-γ-I	86.5% reduction in ω, α genes and 74% reduction in γ-gliadin gene promoter	Gene silencing	(143)
3.	RNAi	All gliadin proteins	Use of specific inverted repeat sequences and hairpin construct	Gene silencing	(144)
4.	RNAi	α-gliadins	Specific genetic deletion of storage protein fraction	Gene silencing	(145)
5.	RNAi	HMW-glutenins	Reduced HMW-glutenin content in wheat	Gene silencing	(146)
**(B) Non-transgenic**					
1.	CRISPR/Cas9	Gliadin proteins, particularly α-gliadins	Mutant lines had reduced gliadin contents	Reduction in α-gliadins	(147)
2.	Breeding	Gliadin proteins, particularly α-gliadins	Breeding wheat with CD specific non-immunogenic wild relatives of wheat	Reduction in α-gliadin epitopes	(152, 153)
3.	Wheat deletion lines	ω, γ gliadins, and LMW-glutenins on short arm of chromosome 1D	Reduced ω, γ gliadins, and LMW-glutenins in wheat	Reduction in CD-eliciting epitopes	(151)
4.	Wheat deletion lines	α-gliadins on short arm of chromosome 6D	Reduced α-gliadins in wheat	Reduction in CD-eliciting epitopes	(148)
5.	Wheat deletion lines	Mutant line lacking *Gli-D2*	Reduced α-gliadins in wheat	Reduction in CD-eliciting epitopes	(150)
6.	Wheat deletion lines	α-gliadins on short arm of chromosome-6	Reduced α-gliadins in wheat	Reduction in CD-eliciting epitopes	(149)
**(C) Microbial degradation**					
1.	*Aspergillus niger, Flavobacterium meningosepticum, Sphingomonas capsulate*, and *Myxococcus xanthus*	Gluten	Reduction by gluten hydrolysis through enzyme prolyl endopeptidases	Reduction in gluten content	(155, 156)
**(D) Probiotics supplementation**					
1.	*Lactobacillus sanfranciscensis* LS40 and LS41 and *L. plantarum* CF1		Improved nutritional content by increasing availability of free Ca, Mg and Zn in gluten-free bread	Enhanced nutrient absorption	(164)
2.	VSL#3	Gluten	Digestion of proline-rich gluten peptides through bacterial proteases	Reduction in gluten content	(161, 162)
3.	*L. acidophilus, L. sanfranciscensis*	Gluten	Degradation of ω-gliadins and HMW-glutenins	Reduction in gluten content	(165)
4.	*Bifidobacterium bifidum* CECT 7365	Gut mucosa	Exerted protective effect on gut mucosa by increasing production of MCP-1 and TIMP-1	Beneficial to gut mucosa	(157)
5.	*B. bifidum* IATA-ES2, *B. longum* ATCC 15707	Gut Health	Reduced levels of IL-12 and IFN-secretion in CaCo2 cell cultures	Reduction in CD immunogenicity	(166)
6.	*B. longum* CECT 7347, *B. bifidum* CECT 7365	Gut Health	Reduced TNF-α and IFN-γ and increased IL-10 production	Reduction in CD immunogenicity	(167)
7.	*B. breve* B632, BR03	Gut Health	Restored normal gut microflora in 40 children suffering from CD	Reduction in CD immunogenicity	(158)
8.	*B. lactis*	Gut Health	Prevented cellular damage of epithelial cells by preserving tight junctions	Reduction in CD immunogenicity	(168)
9.	*Bifidobacterium* spp.	Gut Health	Reduced inflammatory response in CaCo-2 cells by lowering the production of IL-1β, NF-kappaB, and TNF-α	Reduction in CD immunogenicity	(169)
10.	*B. longum* CECT 7347	Gut Health	Increased villus width, enterocyte height & IL-10 levels; reduced gut mucosal inflammation in animal model	Reduction in CD immunogenicity	(170)
11.	*B. infantis* Natren Life Start (NLS)	Gut Health	Improvement in digestive symptoms in CD patients	Reduction in CD immunogenicity	(163)
12.	*L. casei* ATCC 9595	Gut Health	Reduced TNF-α in HLA-DQ8 transgenic mice	Reduction in CD immunogenicity	(171)
**(E) Gluten sequestering polymers**					
1.	Poly (hydroxyethyl methacrylate-co-styrene sulfonate) (P[HEMA-co-SS])	Gluten	Sequesters gluten in small intestine, decreases formation of CD-eliciting gluten peptides and reduces the severity of immune response	Prediction^*^	(172, 173)
2.	Ascorbyl palmitate	Gluten	Decreases gliadin availability and deamination by TG2	Prediction^*^	(174)
**(F) Vaccination**					
1.	Nex Vax® Vaccine (ImmusanT, Cambridge, USA)	HLA-DQ2	Builds up resistance against gluten peptides	Clinical Trial	(175)
**(G) Enzymatic**					
1.	*Prolylendopeptidase* from *Flavobacterium meningosepticum*	Gluten	Detoxifying immunogenic peptides	Reduction in gluten content	(176)
2.	*Cysteine proteinase* EP-B2 from barley	Gluten	Gluten hydrolysis and degradation to small non-immunogenic peptides	Reduction in gluten content	(177, 178)
3.	ALV003 (Alvine Pharmaceuticals, San Carlos, CA, USA), consisting of barley *cysteine proteinase EP-B2* and *Sphingomonas capsulate* PEP	Gluten	Drug reduced gliadin-induced T-cell response and harmful effect on intestinal epithelial cells in patients with CD	Clinical Trial	(179, 180)
4.	*A. niger prolyl-endopeptidase* (AnPEP) and amaranth flour blend (AFB)	Gluten	Reduction in immunoreactive gluten content in wheat dough	Reduction in gluten content	(181)
5.	AnPEP	Gluten	Production of gluten free foods below 20 mg gluten/kg food	Reduction in gluten content	(182)
6.	AnPEP	Gluten	Degradation of ω-gliadins and HMW-glutenins	Reduction in gluten content	(165)
7.	AnPEP	Gluten	Enzyme degraded the immunogenic proline-rich residues in gluten peptides of wheat flour by 40%	Reduction in gluten content	(183)
8.	Engineered endopeptidase (Kuma030)	Gluten	Reduced gliadin content of foods below threshold value of 20 mg/kg	Reduction in gluten content	(184)
9.	Proteolytic enzymes from *Nepenthes* spp.	Gluten	Low gliadin content due to gliadin digestion and reduced IELs	Reduction in gluten content	(185)
**(H) Anti-inflammatory drugs**					
1.	Glucocorticoids-Prednisone, Fluticasone propionate	B and T-cell proliferation	Improvement in weight, sugar absorption, small intestinal enzymatic activity and intestinal histology in CD patients and reduction in lymphokine levels	Prediction^*^	(186, 187)
2.	Anti-interferon-γ (infliximab, certolizumab and adalimumab) and Anti TNF-α (itolizumab)	Targets activation of metalloproteine-ases (MMPs)	MMPs induces pre-inflammatory response, blocking them reduces inflammation	Prediction^*^	(188–190)
3.	Anti-interleukin 15	Cytotoxic T lymphocytes	Reduction in intestinal damage caused by T-cells in mouse models	Prediction^*^	(175)
4.	Interleukin 10	Gliadin induced T-cell activation	IL-10 used for treatment of Th1 mediated autoimmune disorders	Prediction^*^	(191)
**(I) Modified Gluten**					
1.	Genetically modified gluten	Gluten	Reduction in T-cell activation; Transamidation by attaching lysine methyl ester to glutamine residue of α-gliadin	Prediction^*^	(192)
2.	Chemo-enzymatic-Microbial *transglutaminase*	Glutamine in gluten proteins	Transamidation of glutamine with n-butylamine under reducing conditions	Prediction^*^	(193)
3.	Enzymatic-Microbial *Chymotrypsin* and *transglutaminase*	Gluten proteins	Transpeptidation reaction-Binding of valine or lysine to gluten proteins	Prediction^*^	(194)
**(J) Transglutaminase inhibitors**					
1.	Cystamine and cysteamine	Cystamine oxidizes two vicinal cysteine residues on TG2, whereas, cysteamine acts as competitive inhibitor for transamidation reactions catalyzed by TG2	Can reduce the activity of TG2	Prediction^*^	(195)
2.	Inhibitor Zed1227		Reduce the activity of TG2	Prediction^*^	(196)
3.	Reversible T2G inhibitors: •Synthetic polymer poly (hydroxymethyl methacrylate- co- styrene sulfonate) •Anti-gliadinIgY •Dihydroisoxazo-les •Cinnamoytriazo-le •Aryl β-aminoethyl ketones	Covalent modification of enzyme	GTP and GDP are mostly used to inhibit TG	Prediction^*^	(192, 197)
**(K) Others**					
1.	Modulation of tight junctions by AT1001 peptide, Larazotide acetate from *Vibrio cholera*	Zonulin	Antagonizes zonulin activity and prevents opening of intestinal epithelial tight junctions. Inhibits paracellular movements of gluten peptides across tight junctions in intestine	Prediction^*^	(198–200)
2.	Blocking HLADQ2 or HLADQ8 by HLA blockers	HLADQ2/ HLADQ8	To avoid presentation of gliadin peptides by antigen-presenting cells to CD4+ T cells	Prediction^*^	(138)
3.Blocking of Interleukin-15 (a)	Anti-IL-15 monoclonal antibodies	IL-15	Neutralizes enterocyte apoptosis and down-regulates adaptive immune response in lamina propria	Prediction^*^	(138, 201, 202)
(b)	AMG 714 (Anti-IL-15 monoclonal)	IL-15	Reduces immune response to gluten intake	Clinical Trial: Phase 2	(203)
4.	Antagonist of ω-secalin gene (Decapeptide QQPQRPQQPF)	K562(S) cells	Prevents agglutination of k562 cells and hence preventing cell mucosa immune activation	Prediction^*^	(154)
5.	Tolerogenic nanoparticles	Antigen presentation w/o co-stimulation on synthetic antigen presenting cell. Anti-FAS ligand antibody delivers apoptotic signal	Direct action on effector T cells; inhibition of CD4+ and CD8+ T-cell activation	Prediction^*^	(204–206)

* *Predictions represent results based on experimental lab studies and no clinical trials*.

Use of therapeutic vaccine “NexVax2” developed by the biotechnology firm “ImmuSanT” is at the clinical trials stage. The vaccine consists of three gluten peptides and is supposed to induce tolerogenic response in CD patients by building up resistance against gluten peptides (175). “NexVax2” has recently been granted Fast Track designation by the U.S. Food and Drug Administration on January 2, 2019 (207). Other methods include use of gluten sequestering polymers such as poly (hydroxyethyl methacrylate-co-styrene sulfonate) (172, 173) and ascorbyl palmitate (174) which sequester gluten peptides and reduce their availability for subsequent immunogenic reactions. Chemo-enzymatic-microbial transglutaminase (193) and enzymatic-microbial chymotrypsin and transglutaminase (194) have also been used to lower gluten content in wheat flour. Various enzymes used for gluten hydrolysis involve prolylendopeptidase from *F. meningosepticum* (176), cysteine proteinase EP-B2 from barley (177, 178), ALV003 (Alvine Pharmaceuticals, San Carlos, CA, USA), consisting of barley cysteine proteinase EP-B2 and *S. capsulate* prolyl endoprotease (179, 180, 208), *A. niger* prolyl endoprotease (AnPEP) (165, 182, 183), and engineered endopeptidase (Kuma030) (184).

Other strategies involve modulation of tight junctions by targeting zonulins (198–200); blocking of IL-15 using anti-IL monoclonal antibodies (138, 201–203), blocking HLADQ2 or HLADQ8 by HLA blockers (138), use of genetically-modified gluten with modified glutamine residues (192), use of transglutaminase inhibitors (192, 195–197) and use of tolerogenic nanoparticles (204–206). Use of anti-inflammatory drugs (186, 187), anti-IFN-γ, anti-TNF-α (188–190), and anti-IL-15 agents (175) have also been suggested in reducing CD toxicity.

Many among these approaches are just hypotheses or predictions based on experimental data or some are at clinical trial levels. Also, none of these technologies ensure complete removal of CD-eliciting gluten epitopes from wheat and its safe consumption to avoid immunogenic response, but a good amount of research is being done in various spheres to find out promising strategies and alternatives to make gluten free foods in near future. Various strategies for lowering CD epitopes in wheat are given in Table 3.

#### Effect of Lowering CD Epitopes on Bread Quality

Gluten is a structural protein in wheat and is essential for dough making and preparing good quality baked products. Therefore, obtaining wheat varieties with reduced CD epitopes is a technological challenge due to the inability of gluten free flours to form dough with desired strength and visco-elastic properties (209). Furthermore, the baking and bread-making qualities of such flours may get adversely affected.

In a study by Van den Broeck et al. (148), altered dough mixing properties and dough rheology were observed in the hexaploid wheat cv. Chinese Spring deletion lines which were found to be less immunogenic as a result of missing short arm of chromosome 6D containing α-gliadin genes. The dough had reduced elasticity and higher stiffness. In contrast, the technological properties of wheat were retained in the deletion lines of wheat created by removing ω, γ-gliadins and LMWGS from the short arm chromosome 1D. Similarly, Piston et al. (210) found that no major effect on dough gluten strength was observed when γ-gliadins in bread wheat were down-regulated. In another study, Van den Broeck et al. (151) found increased dough elasticity and deteriorated dough quality in wheat having a reduced number of T-cell stimulatory epitopes (ω, γ-gliadins, and LMWGS) in short arm of chromosome 1D. Gil-Humanes et al. (211) reported that down-regulation of CD eliciting gliadin epitopes in different bread wheat varieties by RNAi provided flours with increased stability, different texture, less extensibility and less stickiness in comparison to dough from wild wheat. In a recent study by Zhang et al. (146), the silencing of HMWGS in wheat through RNAi and post-transcriptional gene silencing significantly reduced dough properties, wet gluten content, sedimentation value, and stability time of flour.

### NCGS

In 1978, Ellis and Linaker (212) described a case with diarrhea and abdominal pain in the absence of CD (without any histological duodenal lesions or damage in the lining of small intestine) that improved with the elimination of gluten from the diet. Similarly, Cooper et al. (213) observed abdominal pain, diarrhea, and normal duodenal histology in patients. Their condition improved with intake of diet free from gluten but symptoms reoccurred following the gluten challenge. Since then, the terminology, NCGS is used where intestinal permeability and adaptive immune system have a less pronounced role than in CD (214).

NCGS is caused by gluten and carbohydrates present in wheat mainly the FODMAPs, ATIs and wheat-germ agglutinin (117, 215, 216). ATIs are capable of triggering TLR 4 present on myeloid cells, leading to the release of proinflammatory cytokines (21). The major symptoms in NCGS patients include abdominal bloating and pain in the upper or lower abdomen, diarrhea, nausea, aphthous stomatitis, and changing bowel habits. Other non-gastrointestinal symptoms include foggy mind, fatigue, tiredness, lack of well-being, headache, depression, anxiety, joint/muscle pain, numbness in legs or arms, and skin rash/dermatitis (214, 217, 218). These symptoms disappear when gluten is removed from the diet (219) and overlap with those of irritable bowel syndrome (220). Some of these symptoms are also present in CD but both these conditions differ in their genetics and immunological responses. NCGS can be separated from CD in terms of HLA index, specific immunological response; and the structure and function of small intestinal mucosa (221, 222). The differences between CD, NCGS and wheat allergy are given in Table 4.

**Table 4:** Differences between CD, NCGS, and wheat allergy.

**Terms**	**CD**	**NCGS**	**Wheat allergy**
Definition	Autoimmune disorder due to intolerance to gluten proteins	Disorder due to gluten proteins, FODMAPS in food, ATIs in wheat. Different from CD and wheat allergy	Allergic reaction to wheat containing foods through food ingestion, contact, inhalation of flour dust
Reaction time	Slow (30 min to 24 h)	Slow (several hours)	Immediate
Epidemiology	Affects roughly 1% of population	Affects 0.6–6% of population	0.5–9% in children, 0.2–1% in adults
Antigen	Gliadins from gluten	Gluten proteins, ATIs, FODMAPS	ATIs, Gliadins, Peroxidase, Thiol reductase
Immune response activation	Both innate and adaptive immune response	Innate immune response	IgE mediated immune response
	Deamination by enzyme T2G	No such involvement of enzyme studies till date	
	Activation of inflammatory cytokines like IFN-γ	No such activation	
Hallmark	Lymphocytic duodenosis	Functional dyspepsia, Lymphocytic duodenosis in some cases only	Type I and Type IV hypersensitivity
	IEL levels increased->25/100 enterocytes	In Functional dyspepsia, no increase in IELs but increase in duodenal eosinophils	
HLA genotyping (HLA DQ2 and DQ-8)	Present in 95% of patients	Present/absent, 50% of patients	Not used
Serological analysis			
Anti-T2G antibody	Positive	Negative	No need
Anti-EM antibody	Positive	Negative	No need
Anti-gliadin antibody	Positive	Positive	No need
Anti-deaminated gliadin peptide	Positive	Negative	No need
Ig E antibodies	No need	No need	Positive (Wheat specific IgE)
Histological response	Villous atrophy with crypt hyperplasia	Mildly inflamed mucosa, activated circulating basophils	None
Duodenal biopsy	Positive, MARSH type 3	Negative, MARSH type 0 or 1	No need
IBS indication	Absent/ less prevalent than NCGS	Overlapping with IBS, with 48% of patients affected	Absent
Skin Prick test	No need	No need	Positive
Symptoms Intestinal	Chronic diarrhea, weight fluctuation, weakness, fatty stools, abdominal bloating	Diarrhea, weight loss, gas	Diarrhea and vomiting immediately after wheat ingestion
Extra-intestinal	Infertility, thyroiditis, muscle cramps, delayed growth, iron deficiency anemia	Glossitis, leg and arm numbness, headache, anemia, dermatitis, tiredness, foggy mind, depression, anxiety	Exercise induced anaphylaxis, Atopic dermatitis, Urticaria, Chronic asthma and rhinitis.
GFD	Effective control	Partially effective	Partially effective
Overlap with other autoimmune illness	Increased prevalence Type I diabetes-5% Autoimmune thyroiditis-19%	Not so common Type I diabetes-not found Autoimmune thyroiditis-1.3%	–
Treatment	Following GFD	Avoidance of gluten, FODMAPS in diet (Gluten challenge)	Avoidance of wheat (contact, ingestion, inhalation)

#### Pathogenesis of NCGS

Although CD and NCGS share a common feature of gluten sensitivity; they differ with respect to immune response initiated during their onset. Unlike CD, NCGS is not related to autoimmune process involving adaptive immune response leading to intestinal epithelial cell damage (223). Many studies have thrown light on the underlying mechanisms involved in the pathogenesis NCGS but no confirmed mechanisms are still known. Sapone et al. (224) observed normal intestinal permeability and increased levels of IELα, IELβ, and TLR-2 expression in 26 NCGS patients in comparison to CD patients and controls. Different studies have shown the involvement of only intestinal innate immune system in NCGS as evident by the increase in TLR2, TNF-α, IL-8, and IL-12 expression (21, 225, 226). However, in some studies, an increase in anti-gliadin Ig antibodies and IFN-γ expression has also been observed (227–229). Uhde et al. (230) showed that individuals with NCGS displayed increased serum levels of intestinal fatty acid and lipopolysaccharide-binding proteins along with elevated levels of soluble cluster of differentiation 14 (CD14), all of which declined in response to the elimination of gluten from food. Carroccio et al. (227) reported basophil activation in 66% of patients with NCGS and found it to be associated with intraepithelial lymphocytosis of duodenum and infiltration of eosinophils in duodenum and colon. Some NCGS patients showed increased TLR2 levels in comparison to CD, and had dysbiosis similar to that observed in inflammatory bowel disease (231). Furthermore, in a study by Junker et al. (21), it was observed that wheat ATIs act as triggers of the innate immune response in intestinal monocytes, macrophages and dendritic cells by activating the TLR4-MD2-CD14 complex and eliciting the release of proinflammatory cytokines in cells from celiac and non-celiac patients.

#### Diagnostic Parameters for NCGS

At present, there are no known specific serological markers for NCGS. Unlike CD, diagnosis of NCGS is not dependent on the patient's response to different antibodies or biopsy analyses. Only anti-gliadin antibody can be used to test NCGS, while anti-TG2 and anti-EM antibodies are found to be negative in these patients (232, 233). Various studies have reported that 25–50% of NCGS patients have serum anti-gliadin antibodies, mainly IgG (225, 227, 234). Volta et al. (235) reported an increase in the levels of IgG antibodies specific to gliadins in NCGS patients. NCGS is diagnosed clinically on the basis of response to gluten free food, followed by gluten challenge (214, 236, 237). In a study by Catassi et al. (236), NCGS specific symptoms disappeared in patients after the removal of gluten from the diet and reappeared during oral gluten challenge performed after at least 3 weeks of gluten-free food in a double-blind placebo experiment. Gluten challenge is generally initiated once the patient's symptoms are reasonably under good control. The clinical response after gluten challenge might be variable but usually overlaps with symptoms of CD to a large degree (225, 238). Histological findings indicate duodenal biopsy to be between MARSH type 0-1 and there is also infiltration of CD3+ IELs (ranging from 25 to 40 IELs per 100 enterocytes) but not to an extent as seen in CD patients. Moreover, some levels of circulating basophils are also present (224, 227, 238–240). Verdu et al. (241) carried out a study on gluten sensitive transgenic mice with HLA-DQ8 genes and found that gliadin exposure resulted in the activation of innate immunity without any intestinal atrophy in these mice. However, Bucci et al. (242) carried out studies on NCGS patients and found that gliadin exposure did not result in the activation of mucosal inflammation or basophil production.

### Wheat Allergy

Wheat allergy is characterized by IgE and non-IgE mediated immune response resulting in allergic reaction in certain individuals upon the uptake, contact, or inhalation of foods containing wheat (243, 244). IgE mediated allergic responses occur immediately after food consumption, are food-specific and reproducible (245) and result from the release of histamine, platelets activator factor, and leukotrienes from mast cells and basophils (246, 247). These allergic reactions can affect skin, respiratory or gastrointestinal tract and are characterized by TH2 lymphocytic inflammation which leads to the production of IL-4, IL-5, and IL-13; and causes B cells to produce IgE antibodies specific to certain foods (215, 248, 249). Genetic characteristics of individuals as well as environmental factors play an important role in the onset of such immune response (250, 251). Non-IgE mediated allergic responses are characterized by chronic infiltration of eosinophils and lymphocytes in the gastrointestinal tract. Different types of wheat allergies include:

#### Food Allergy

Food allergy is caused by the intake of wheat which triggers the IgE mediated immune response in certain individuals. Different components of wheat can cause food allergy, *viz*., ATIs, non-specific lipid transfer protein, gliadins, and LMWGS (40, 252, 253). Food allergy results in the development of different symptoms in patients, *viz*. urticaria, stomach cramps, asthma, allergic rhinitis, abdominal pain, vomiting and atopic dermatitis (42, 254). Remedy for food allergy includes the avoidance of wheat in the diet.

#### WDEIA

WDEIA is a type of food allergy which occurs after wheat ingestion followed by physical exercise but is not triggered by wheat ingestion alone. WDEIA was reported only few decades ago in 1985 (255) and its symptoms include anaphylactic reactions ranging from urticaria, angioedema, dyspnoea, hypotension, collapse, and shock. In wheat, ω5-gliadin, and HMWGS have been reported to be the major allergens that contribute to WDEIA (256–259). WDEIA is also induced by non-steroidal anti-inflammatory drugs, alcohol, and infections. The remedy suggested for this is to avoid having gluten rich diet before physical exercise (260). The exact mechanism of WDEIA is still not clear, but exercise enhances gastrointestinal osmolarity and permeability to allergens, increases blood flow and induces IgE-mediated mast cell degranulation (261, 262). Increased temperature also causes phosphorylation of tight junction proteins which results in increased absorption of allergens due to mucosal injury (263).

#### Baker's Asthma

Baker's asthma develops after inhalation of allergens, particularly cereal flour dust present in the work environment and affects 0.03–0.24% of bakery workers, confectioners, pastry factory workers, and cereal handlers. It is considered one of the most common types of occupational, cereal-induced allergic asthma and is mediated by IgE antibodies specific to cereal flour antigens: mainly proteins from wheat, rye, barley and rice (264–266). The primary cereal used in bread baking is wheat and it acts as a major allergen in 60–70% of symptomatic bakers (267). Bakers develop asthma as well as rhinitis with increased levels of specific IgE antibodies against flour dust from different sources (268). Incidences of bronchial hyper-responsiveness are also reported in bakery workers suffering from Baker's asthma (269, 270). Genetic factors also play a significant role in the onset of Baker's asthma with respiratory symptoms. In a study by Cho et al. (271) on Korean bakery workers, TLR4 gene polymorphism was found to be responsible for allergic sensitization to wheat flour. Similarly, Hur et al. (272) reported genetic polymorphisms of β2-adrenergic receptors to be responsible for the development of Baker's asthma in the workers exposed to wheat flour. ATIs along with non-specific lipid transfer protein are the major components of wheat resulting in the onset of Baker's asthma (40, 253). Other allergens include wheat peroxidase, thioredoxins, serine proteinase inhibitor, thaumatin-like proteins, gliadins, and LMWGS (41, 273–276).

Proper diagnostic measures for Baker's asthma are not present till date. The only available method is skin prick test using commercially available wheat extracts for serological analyses but this method is less sensitive and non-specific due to different concentration and composition of antigens in different wheat extracts, and implementation of non-standardized methods of preparation (117, 277). Hence, improved and standardized measures for the diagnosis of Baker's asthma are required.

## Is Wheat Safe for Non-celiac Healthy Individuals?

**Figure 5:**
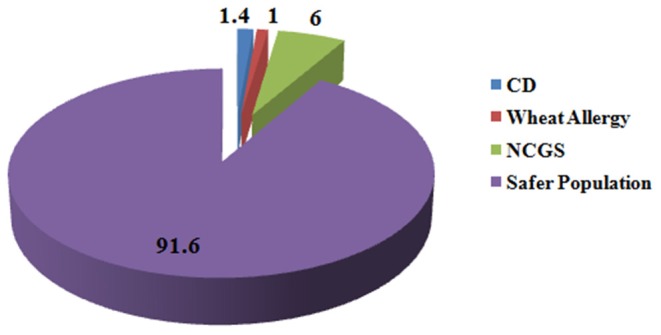
Percentage of people suffering from gluten-related disorders. Figure shows that in total 8.4% of people across the globe suffer from gluten-related disorders and 91.6% of population is safe from these disorders and is non-susceptible for gluten ingestion.

Wheat is the most important cereal crop across the world and is rich source of variety of nutrients such as carbohydrates, proteins, fats, dietary fibers, lipids, B vitamins; minerals such as iron, calcium, zinc, magnesium, sodium, copper, and selenium; and many phytochemicals such as phenols and flavonoids (3). Though a number of diseases and conditions arise from wheat intake in certain individuals who cannot tolerate it but wheat consumption is safe for a wide percentage of individuals who can tolerate it. Globally 1.4% of individuals are reported for CD (53), 0.63–6.0% for NCGS (278), and 0.2–1.0% for wheat allergy (245, 249). Hence, at maximum, only 8.4% population is susceptible to wheat-related disorders and the remaining 91.6% population remains safe for the intake of wheat and should not remove it from diet based on false media coverage (Figure 5).

## Is There Difference Between Diploid, Tetraploid, or Hexaploid Wheat Cultivars in Eliciting CD?

The diploid wheats (AA, 2n = 14; *T. urartu, T. monococcum*) and the tetraploid wheats (AABB, 2n = 28; *T. durum, T. turgidum*) were domesticated by man, about 10,000 years ago (279). Modern bread wheat is allohexaploid (AABBDD, 2n = 42; *T. aestivum*) species arising from hybridization between tetraploid *T. turgidum* having AB-genome and wild diploid species *Aegilops tauschii* having D genome (280). The introduction of D-genome in wheat has improved its bread-making properties (281–283). At genomic level, the genes encoding for immunogenic CD epitopes are located on the short arm of 6A and 6D chromosomes, while the short arm of 6B chromosome is mainly non-immunogenic. The highly immunogenic 33-mer CD epitopic sequence of α-gliadins is located on the 6D chromosome which can initiate a very strong immune response (70, 284). The literature suggests that the hexaploid wheat consists of more immunogenic CD epitopes and elicits a higher immunogenic response in CD patients than diploid and tetraploid wheat due to the presence of highly immunogenic D genome (79). Different studies have shown that tetraploid wheat are less immunogenic than hexaploid wheat (285–287). In one study, Schalk et al. (284) did not detect any 33-mer peptide sequence in two durum wheat cultivars and two emmer cultivars (genome AABB) by using liquid chromatography tandem mass spectrometry (LC-MS/MS) and attributed this to the absence of chromosome 6D. Similarly, Molberg et al. (288) showed absence of 33-mer peptide sequence encoded by α-gliadin genes in diploid einkorn (including *T. monococcum, T. uraru*, and *A. speltoides*). Kumar et al. (289) studied 34 tetraploid and hexaploid wheat varieties for their gliadin content and immunoreactivity with immunoglobulins (IgA) of CD and found tetraploid wheat varieties to be less immunoreactive than hexaploid wheat varieties. Ozuna and Barro (290) found in their study that durum wheat varieties tend to have lower gluten protein and CD eliciting epitopes in comparison to tetraploid and hexaploid wheat varieties.

Studies by De Vincenzi et al. (291); Pizzuti et al. (292), and Vincentini et al. (293) demonstrated that *T. monococcum* was non-immunogenic w.r.t. CD, while others studies showed that immunogenicity of diploids still exists, but is very less in comparison to hexaploid wheat varieties (79, 285, 286, 294, 295). On the contrary, in other studies, no difference in the immunogenic potential of diploid, tetraploid, and hexaploid wheat was observed (296–298). Ozuna et al. (286) reported *A. tauschii* to be highly immunogenic, while Escarnot et al. (295) showed lower immunogenicity of *A. tauschii* in comparison to bread wheat. Vaccino et al. (299) found the presence of 13 immunogenic CD eliciting peptides sequences in *T. monococcum*, thereby indicating that diploid wheat has the potential to trigger CD.

## Is Breeding Responsible in Increasing CD Epitopes in Wheat?

At present, 95% of the wheat grown globally is hexaploid (utilized in bakery) and the remaining 5% is tetraploid (utilized for pasta making) (300). Studies have claimed that over the decades, the genetic improvement of wheat NexVax2 through breeding to improve yield, plant height, disease resistance, adaptation to climate changes, and bread-making characteristics by modifying wheat proteins particularly gluten content, may have led to a higher immunogenicity of wheat and therefore higher incidences of CD (279, 301).

Gluten constitutes 60–75% of total wheat proteins and is highly desirable in the food industry and is mainly responsible for imparting the desirable strength to the dough and contributes to its viscoelastic nature (302, 303). In addition, increase in wheat consumption, use of gluten in food processing, and consumption of processed foods has been seen over the years (304, 305). The preference of wheat varieties with higher gluten content has always been desirable but in spite of being an important wheat protein, gluten acts as a main source of immunogenic peptides triggering CD in certain individuals.

Many studies have evaluated the role of breeding on the immunogenicity of wheat. Kaur et al. (306) studied variation in CD-eliciting epitopes of α-gliadins protein sequences in Indian wheat cultivars and found modern varieties (1971–2011) having a higher amount of intact T-cell stimulatory epitopes than old wheat varieties (1905–1970) which had comparatively higher variant epitopes. Van den Broeck et al. (307) analyzed the potential toxicity of several hexaploid wheat varieties including 36 modern and 50 landraces using Glia-α9 and Glia-α20 antibodies and found higher amounts of Glia-α9 epitopes in modern varieties. This suggests that modern wheat breeding practices may have led to a higher number of CD-triggering epitopes. De Santis et al. (308) explored the effect of breeding in the twentieth century in Italy and found an increase in gliadin and glutenin epitopes in modern durum wheat varieties. However, Kasarda (279) observed that breeding of wheat to improve its baking quality and dough strength did not influence antibody A1-G12 reactivity in old, mid and new wheat varieties from the 20^th^ and twenty-first centuries in the United States of America. Also, the comparison of different landraces and wheat varieties did not show any significant difference in A1-G12 reactivity. But in some other studies, modern wheat varieties were found to have a reduced number of CD epitopes in comparison to old wheat varieties or landraces. Ribeiro et al. (298) showed that *T. aestivum* spp. *vulgare* landraces had higher reactivity to R5 antibody and presented higher amount of potential CD immunostimulatory epitopes than modern varieties; inferring that breeding practices were not responsible for the increase in CD immunostimulatory epitopes. Gelinas and McKinnon (287) found that the traditional wheat line available in the nineteenth century showed the highest reactivity to G12 antibody. Similarly, Schalk et al. (284) found that spelt cultivar Ober-kulmer had the highest amounts of the 33-mer peptide (523.4 μg/g flour), while other spelt cultivar Franckenkorn (353.9 μg/g flour) showed no significant difference from the common wheat cultivars used in the study. Similarly, no significant difference was observed in A1-G12 reactivity of tetraploid and hexaploid wheat varieties in a study by Escarnot et al. (295). Malalgoda et al. (309) quantified Glia-α9 and Glia-α20 by mass spectroscopy in 30 wheat varieties released between 1910 and 2013 in North Dakota but could not find any trend in the immunogenicity of these wheat varieties. Some other studies have also reported comparatively higher toxicity in ancient wheat varieties in comparison to modern wheat varieties. Prandi et al. (310) analyzed gluten peptides in old and modern *Triticum* varieties, using Liquid Chromatography Mass Spectroscopy (LC-MS) and found a significantly higher amount of CD-eliciting immunogenic peptides in old varieties than modern varieties. They concluded that old wheat varieties have the potential to trigger CD and are thus not safe for CD patients. Similarly, Gregorini et al. (311) and Colomba and Gregorini (312) found a higher percentage of immunogenic α-gliadin epitopes in ancient durum wheat varieties namely Graziella Ra and Kamut in comparison to modern wheat varieties and advised CD patients to avoid consuming these wheat varieties. All these studies suggest that there is no fixed trend in the results obtained due to immense variation in the immunogenic potential of different wheat varieties: old, modern or landraces. Thus wheat breeding did not cause any increase in CD toxicity (296, 310, 313, 314). This can be attributed to the fact that the genetic improvement of wheat through breeding has mainly focused on glutenins, which are mainly responsible for dough strength and are less immunogenic in comparison to gliadins. Ozuna and Barro (290) found in their study that breeding has infact contributed to the decrease in gliadin and total gluten content but not glutenin content in different wheat varieties belonging to *Triticeae*.

## Gluten Free Diet (GFD)

At this point of time, therapies for CD patients are non-existent. The only solution to the problem is a stringent lifelong GFD. GFD means the absence of gluten or prolamine proteins from wheat, rye, barley and oats in natural as well as in processed foods (315–317). The limit of gluten in GFD has been set to 20 ppm by Codex Alimentarius Commission for International Food Standards of Food and Agriculture Organization of the United Nations [FAO]/World Health Organization (318). This cut-off limit is followed in many countries including Spain, Italy, UK, Canada, and USA but countries like Australia, New Zealand, and Chile have locally set the cut-off limit to 3 ppm, while Argentina has set the limit to 10 ppm (319).

In a double blind, placebo controlled study by Catassi et al. (320), it was observed that significant reduction in intestinal mucosal villous height/crypt depth ratio occurred upon intake of 50 mg gluten per day for 3 months but in a study by Lanzini et al. (321), intake of <10 mg gluten daily did not cause any considerable histological changes in the intestinal mucosa. Laurikka et al. (322) compared gastrointestinal symptoms in untreated, treated with GFD and healthy controls and observed higher diarrhea, indigestion, and abdominal pain in untreated CD patients than those on GFD and controls. Also, a very good response was observed in patients on GFD during a long-term follow-up. Akobeng and Thomas (323) observed that GFD with gluten concentration lower than 20 ppm required an intake of <50 mg gluten daily to ensure adequate safety.

### GFD: Challenges

Correct quantification of gluten, its source, and proper labeling are necessary for the normal health of CD patients. Following a life-long GFD can be very challenging due to the presence of small amounts of hidden gluten in processed food products as contamination of foods is possible at different stages from farm to fork. Cross-contamination of GFD from gluten based foods can occur from grains from adjacent fields; harvesting, storing, and processing grains on shared equipment; making products on same equipment; food handling by unaware employees in various restaurants, and lack of awareness among people in general. Other challenges related to GFD are higher cost, less palatability, less availability, and less reliability in developing countries (324–326). Also, the likelihood of low income patients in developing countries to go on GFD is very low (327). For CD patients, eating out and traveling are major challenges because of poor availability of GFD and fear about gluten contamination. Producing low gluten-immunogenic wheat and food products will involve a great deal of efforts, as well as scientific and technological inputs. Educating people about GFD, promoting research and development for the production of cost-effective GFD and providing country-wide infrastructure are key steps to manage CD and gluten intolerance (328–330). This will help CD patients to follow a life-long GFD and will help to improve their quality of life.

### Methods for Detection of Gluten in Food

The fast growth of CD diagnosis has sparked the investigation for the reliable methods for gluten estimation. Several methods have been proposed for gluten estimation in different food and food products. The most widely utilized method is enzyme-linked immunosorbent assay (ELISA); developed by utilizing monoclonal antibodies against toxic gluten peptides. Mainly, two monoclonal antibodies based (R5 and G12) ELISA kits are commercially available (331, 332). ELISA R5 Mendez Method has been approved by 2015 Codex Alimentarius Commission. In addition, polyclonal antibodies against gluten peptides have been prepared for potential use in ELISAs (333). For increasing the sensitivity of antibody based detection to picogram level, modifications like adsorption of food sources on small latex particles, fluorescent labeling, and flow cytometry have been investigated (334).

LC-MS/MS provides an alternative to ELISA based detection method. Protein extraction method has critical importance for efficacy of LC-MS/MS based methods (335, 336). Digestion of proteins with digestive enzyme or peptic-tryptic/chymotryptic treatment is required for simulating human gastrointestinal digestion of wheat products (337). For the adoption of this technology, the pre-requisite step is the identification of robust and sensitive CD triggering peptide markers as well as comprehensive and well-annotated sequence databases. GluPro V1.0 database of gluten proteins comprises of 630 discrete and unique full-length protein sequences (338) and is an alternative to the existing Viridiplantae database. LC-MS/MS has been documented to distinguish among different cereals and detect cereal contamination in different commercial flours (335, 339). Reverse phase-high performance liquid chromatography coupled with matrix-assisted laser desorption ionization time of flight mass spectrometry has been reported to assess prolamins from wheat (340). Peptide immobilized pH gradient-isoelectric focusing separation, coupled with LC-MS/MS, has been documented for detecting cereal contamination from beer (341). Two-dimensional electrophoresis and gel-permeation high performance liquid chromatography with fluorescence detection have also been reported for the detection of gliadins and glutenins (342, 343).

In order to increase the speed, sensitivity and decrease the cost of current methods, *viz*., ELISA and LC-MS/MS; several alternative electrochemical immunoassays like electronic tongue (e-tongue), nanorods, and immunochips have been proposed. The microfluidic e-tongue capable of detecting as low as 0.005 ppm of gliadin in foodstuffs has been investigated (344). The competitive and disposable amperometric immunosensor based on gliadin-functionalized carbon/nanogold screen-printed electrodes was developed for rapid gluten detection in processed food samples (345). The gliadin-immunochips, based on electrochemical impedance spectroscopy transduction method, capable of detecting 0.5 ppm of gliadin in beers and flours have been reported (346).

## Conclusion

Wheat is one of the most important cereals and with the increase in incidences of wheat related diseases like CD, NCGS, it is imperative that these challenges are addressed now. Patients with CD must avoid foods containing gluten and should strictly follow GFD; patients with wheat allergy should avoid contact with wheat in any form; and patients with NCGS should also adhere to GFD. Much research advancements have happened in the diagnosis of CD and wheat allergy, but not in case of NCGS. Therefore, it is necessary to understand the underlying mechanism of pathogenicity in case of NCGS to develop more sensitive diagnostic markers. The fundamental understanding of the mechanisms of disease relevant pathways may come from the analysis of genome and gene expression.

More research and infrastructure are needed for the development of low-gluten wheat, and ultimately food products. This also requires changes in industrial food processing methods because low gluten content in wheat and its products would be an important trait for its commercialization in future. Plant breeding and biotechnological approaches could be used to make CD-safe wheat and food products with reduced immunogenic gluten fractions. Transferring genes containing less immunogenic CD epitopes from wild relatives of wheat to existing high yielding wheat cultivars can be done by using breeding and biotechnological approaches.

Nevertheless, only a small percentage of global population is affected by these wheat related disorders, hence, opting for GFD to improve well-being by the remaining population without any medical recommendation is an unhealthy option; wheat being a nutrient and dietary fiber rich cereal.

## Author Contributions

MG drafted the manuscript layout and helped in overall supervision during manuscript preparation. NS, SB, and SK collected the literature and wrote the manuscript. MG, VC, SS, PK, and AK helped in manuscript and references editing. NS prepared the figures. NS and SB prepared the tables.

### Conflict of Interest

The authors declare that the research was conducted in the absence of any commercial or financial relationships that could be construed as a potential conflict of interest.
